# Stomatitis

**Published:** 1891-12

**Authors:** V. A. Latham


### Stomatitis—Especially with Reference to Dentition and Dental Surgery.

BY V. A. LATHAM.

Read before the Students Dental Society (U. of M.)

Under this heading we will notice some points of interest connected directly with children at the period of Dentition, selecting Stomatitis as the most common, and though it usually comes more directly under the care of the general practitioner, it is important that dentists should be well acquainted with it. Unhappily medical men know too little of dentistry and the dentist too little of general medicine to be able to thoroughly examine these cases and to treat them as is necessary, seeing they depend for cure on the attention given by both professions. We of all people should know thoroughly the normal healthy appearance of the oral cavity and all it contains, and should therefore also be immediately able to detect any abnormal or pathological condition that may be presented. In the examination of a mouth for disease, we should cultivate as early as possible a habit of thoroughness; we should not be content with looking at the teeth, but inspect the gums which so often disclose much about the condition of the roots of the teeth by the appearance of the surrounding parts. If any abnormality be present, examine for the cause with every care and then decide on the treatment and remedy, noting at the same time the idiosyncrasy of the patient. Every dental surgeon should be able to form a correct diagnosis of any case he may meet with, though to do this often requires considerable book knowledge and practical experience in hospitals and dental infirmaries. Many of these affections we as dentists are called upon to treat, and others after they are diagnonosed we may hand over to the care of the physician. These cases, no matter whether simple or serious, we should be well acquainted with as we are more likely to see them in their earlier stages, and by immediate interference we may warn our patient and thus induce him to obtain medical or surgical attention and thereby save him from a long and serious, yes, and often a fatal illness. I would here mention the difficulty of the diagnosis of oral tumors and the great caution which should be exercised in examination, also the necessity for a prompt decision. For example, in epithelioma it is better to make a great many mistakes and use surgical interference than let one case of epithelioma go too long. Epithelioma travels by the lymphatics and when it begins in this way, the patient is beyond help. In hardly any case of death from cancer of the stomach has the liver been free from secondary cancer cells, for the reason given above; hence, make an early diagnosis, examine every small sore, ulcer and spot in the mouth, using every precaution in antisepsis for the benefit of the patient and yourself. Before discussing Stomatitis, it is well to understand something of dentition as this disease occurs the oftenest during this period. In older children it often appears as the result of some acute infectious disease, especially measles and scarlet fever; but in these cases it frequently passes on to a more severe form with a fibrinous exudation. We all know the readiness with which most disorders of children are attributed by every class of persons to "the teeth." Every convulsion or intestinal trouble which a child may have is so placed, and accordingly either neglected or even regarded as salutary. The physician's aid is called only when too late. This old standing tradition, still in full force among the laity in spite of the improvement of education, is now most positively contradicted by a large number of medical men of to-day. Teething, they hold is a physiological process, which cannot be the occasion of any morbid symptoms, and everything formerly regarded as such is a delusion, caused by illnesses happening to occur along with them without having anything at all to do with it. It seems questionable whether such a positive denial is altogether warranted, and while granted it has rendered much service in limiting the "diseases of teething," it seems to me that there is a want of moderation in this view. We all know dentition occurs by the growing root of the tooth gradually pushing on the already complete crown and forcing it out of the alveolus after it has come through the overlying gum which has gradually been thinned by increasing pressure. Is it, then, so very unreasonable that this gradually advancing process should exert an irritating action on the dental branches of the fifth nerve and occasion those reflex symptoms, extending not only to the province of the motor, but also that of the vaso-motor nerves. To us, it seems possible, and I for one consider it going too far to deny utterly the possibility of convulsions being caused by the irritation of teething, as is so much done by some lecturers on "diseases of children." Instances have been known where partial contractions of the muscles of the throat and neck were undoubtedly connected with the eruption of a group of teeth, not only in children but adults. It is an indisputable fact that obstinate vomiting intestinal troubles, (diarrhœa) a spasmodic cough may occur. Again, when the incisors are being cut infants seem very prone to catarrh of the bronchial tubes and sometimes catarrhal pneumonia. Enlarged glands in children who are predisposed to glandular enlargements, are well known to be caused by the irritation of aphthous patches, either of the submaxillary when the lower jaw is affected, or the parotid lymphatic glands which receive the lymph from the upper part of the face. These swellings may subside or end in either acute or chronic suppuration. In the latter case successive teeth being cut keep up the source of irritation. Eczema of the face and lichen are usually much worse while a tooth is erupting. The eczema very frequently gets well in the intervals, the face and body being free, until a tooth comes near the surface, and the eczema returns, the papules begin to ooze and crust and I can only explain this by the reflex action from the dental branches of the fifth upon the peristalsis, the vagus or the vaso-motor nerves. We must guard against neglecting the views and experiences of our predecessors, who have done so much in the past for the advancement of our science, with that presumption which has become unfortunately so common with some of the younger members of the profession. The irregularity of teething is so well known as to require no mention here, though the following case may serve to show the necessity of a good education in general medicine and to do away with the idea. Oh, well, I shall not have patients to attend to till they are a year or two old.

Case A.—Aet 13 days. Illness dated from fourth day after birth without apparent cause. The margin of the lower alveolus swollen, red, covered with pus, which oozes out on the slightest pressure as from a sponge. During the last few days both lower central incisors have appeared and been extracted, leaving behind two suppurating cavities. The teeth consisting only of a crown coming to a point below without a root. Prof. Henoch cites a number of cases of extremely premature dentition and similar to the above. He considers it probable that periostitis of the alveolar margin, whether in upper or lower jaw, forces the crown of the tooth out by swelling and exudation inside the alveoli, and regards periostitis to be the primary cause and not any violent extraction of the teeth. It will be a study to discover how this disease of the bone, occurring at birth or so early afterwards, was occasioned in these cases. Prof. Samelsohn cites a case of periostitis of the orbit in a child of 14 days old, and thought the cause of disease (eyeball was enormously protruded) was the first molar which was prematurely forced through, and on extracting the case ended favorably. The tooth showed a well developed crown and the beginning of a root. Prof. Henoch differs as to the cause and believes it was the occurrence of periostitis of the upper jaw, due to unknown causes. Before discussing the affection proper, recall the anatomy and histology of the mouth which is known to you all and then we can understand the disease known as

Stomatitis.—The inflammatory lesions which affect the mouth may be classified as follows: (1) Catarrhal; (2) follicular; (3) aphthous; (4) ulcerative; (5) gangrenous; (6) mercurial; (7) parasitic; (8) syphilitic stomatitis.

(1) Catarrhal stomatitis, synonyms. Simple stomatitis, erythematous; catarrh of the mouth.

Defin.—An acute catarrhal inflammation of the whole or a portion of the mucous membrane of the mouth and tongue, characterized by redness, swelling and disordered secretion—mostly found in infants and children.

Causes.—(a) Dentition—usually about the period of eruption of the milk teeth, between the ages of 6 months and 2 years may appear in conjunction secondary Dentition 6—13 years or even in adult life when erupting the wisdom teeth (Dens Sapientiæ) and through inoculation (Barrett, Cosmos, Oct. 1891.

(b) Local irritants, improper food, abuse of alcohol or tobacco, carious teeth, accumulation of tartar.

(c) Digestive disorders, primary or associated and with general disease.

Pathological Anatomy.—Buccal mucous membrane and tongue, dark red appearance, much swollen, tongue seems too broad to lie between the teeth and its sides marked by them; secretions at first less, then increasing, cloudy mucus covers the cheeks, gums and tongue, causing a coated tongue.

Symptoms.—The onset is marked by a temporary diminution of the secretions of the mouth, while the mucous membrane becomes dry, hot and tumid, and is beset with bright red patches which quickly spread and by coalescence form a diffused blush over the whole of the cavity. The saliva is scanty and tenacious, the sense of taste viliated or lost, mastication and deglutition are painful, the breath offensive owing to decomposition of the secretions and the patient may be annoyed by subjective gustatory sensations of a more or less disagreeable nature. In a few hours the secretions are restored and soon become excessive; the mucous membrane becomes redder and more swollen, and presents deep indentations at the points of contact with the teeth; the epithelium thickened and cloudy in places, and may here be detached, leaving superficial erosions; and finally in severe cases the borders of the gums may ulcerate and the teeth become loosened. As a general rule the disease is usually mild, the symptoms passing away within a few days; though occasionally it may defy treatment for several months. Stomatitis produced by eruption of third molar is particularly painful and continuous, and is characterized by sharp neuralgic pains all over the side of the face and forming ulceration of the tongue and jaws.

TREATMENT.—The first thing to do is to remove the exciting cause, attention to the secretion and diet, a dose of aperient medicine, emollient mouth washes; if an infant, a little limewater in the milk. Chlorate of potash is about the best remedy in doses of from 2—6 grs 3 times a day, and when the mouth can bear it combined as follows:

R. Pot. chlor..........................j drachm.
Tinct. cinchona.....................j drachm.
Muriatic acid dil.......... ........j drachm.
Syrup Mulberries..................jss ounce.
Water to.................. .......vj ounces.

SIG. Half an ounce every 4 hrs for children 8 to 10 years. or some iron and mineral acid given instead. Ice to suck. Remember mouth washes cannot be used for infants unless by the nurse.

Borax, Condy's fluid one-half teaspoonful to a pint, watching the action on the teeth, and if these are loose, do not extract them until you have given them a chance to refix themselves in their sockets, or until it is evident that their presence is prejudicial to the healing of the sores.

In obstinate cases which resist even the continued use of chlorate of potash or even become worse under it, without your being able to find any reason for this peculiarity. Good results may be obtained from the use of Sulphate of zinc (grs. xxiv to 1 ounce or sulphate of copper (grs. xii to 1 ounce); with these paint the affected parts two or three times a day.

II. FOLLICULAR STOMATITIS. Syn. Aphthæ; vesicular stomatitis; croupous stomatitis. Is marked by an eruption of red papules surrounded by inflammatory areolæ, and developing apparently in connection with the mucous glands of the lips and cheek. The points generally soften and burst, leaving small excoriations which soon heal.

III. APHTHOUS STOMATITIS. This offers a certain resemblance to the parasitic stomatitis or "thrush," both in appearance and course, but is less superficial and does not present the same fungus on microscopical examination. It is characterized by the appearance of disseminated whitish or yellow patches of sub-epithelial exudation, which give place to superficial excoriations, and it may be associated with febrile disturbance and digestive disorders. This and the last variety usually appear under the same conditions as the catarrhal form.

The treatment is the same as for the above varieties.

IV. ULCERATIVE STOMATITIS. Syn. diphtheritic stomatitis; gingivitis ulcerosa. This is a more specific and troublesome affection than the catarrhal form, and is marked by a fairly definite localization of its principal lesions and an almost absolute limitation to two eras of life.

ETIOLOGY. Causes are constitutional debility mainly induced by impure air and improper or insufficient food; and the second cause is local irritation in association with the eruption of the permanent teeth. It is almost peculiar to children of the poor between the ages of six and twelve or persons from eighteen to twenty-five. In any case it may assume an epidemic form and perhaps is contagious.

SYMPTOMS. The first sign is fetor of the breath with a sense of heat and tenderness of the gums. The saliva small but gradually increases in quantity, becomes profuse, opaque and offensive. The whole of the buccal membrane becomes red, swollen and hyperæsthetic, the gums very tender, sensitive and bleed easily, the teeth loosen and perhaps fall out. The first part of the gums to be affected is usually near the incisors or cuspids of the lower jaw, the fore part of the gums suffering more than the back, and purulent matter flows from between the teeth on pressure, the cheeks and submaxillary connective tissue become œdematous, this swelling and the increasing pallor often disfigure the face of the child so as to cause anxiety and even lead one to fear it is a case of Noma or Cancrum Oris. Mastication is painful. The left side of the mouth is usually attacked, the gums dusky red or purple and swollen and ulcerated, especially where in contact with the molars, covered with a greyish deposit made up of epithelial debris, pus, blood, etc., and containing numerous bacilli. The tongue and mucous surfaces have white patches or superficial ulcers and these also occur on the palate, fauces and tonsils and are likely to be mistaken for mucous tubercles. It must be noted that the ulceration is not associated with an inflammatory induration of the contiguous tissues as in cancrum oris. In very severe cases this variety may end in mercurial stomatitis.

TREATMENT Is smilar, using nourishing food, fresh air, tonics, caustics and mouth washes.

V. GANGRENOUS STOMATITIS.—Syn. cancrum oris, noma, occurs in squalid, half-starved children after eruptive diseases. This disease is often fatal and rapidly spreads over all parts of the face and is accompanied by fœtor, sloughing which often poisons the child and causes septic pneumonia, or death through exhaustion.

Sansom (4) describes an organism he found in the blood and diseased tissues, which on inoculation into guinea pigs and mice appeared in their blood. The *treatment* consists in actual cautery, caustics, lotions, nourishing food, tonics and surgical interference. Black lotion (calomel 3 grs. to 1 oz. lime water) is very beneficial in the early stage.

VI. MERCURIAL STOMATITIS.—This once common disease is now rarely seen in its more acute forms. It usually occurs during the administration of the drug or in connection with industries involving the use of the metal.

The symptoms are those of stomatitis and mercurial poisoning. The treatment as above; the chlorate of potash often exerts a most magical influence over the local lesions. It may be used in the form of a lotion (20 or 30 grs. to the oz.) and given internally in combination with hydrochloric acid and bark. Sulphurous baths hasten the elimination of the poison from the system.

VII. Parasitic Stomatitis, commonly known as thrush, is caused by the growth of a fungus called the oidium albicans in the buccal epithelium. The fungus consists of fine-jointed and branching stems, some of the buds forming capsules, conidia, or spores, by which the growth is propagated. Its identity has been a matter of doubt, though by some thought to be the oidium lactis, or fermentation plant which turns milk sour; but Grawitz (5) has cultivated it in solutions of sugar and he considers it to be the mycoderma vini, or wine ferment. Rees, who has further investigated it, has named it saccharomyces albicans. Its chief seat is the mouth, but it has been found in the larynx, œsophagus and stomach. One must guard against the inexcusable mistake of confounding thrush with leucoplakia oris. Symptoms. It appears as little whitish spots upon a reddened base about the angles of the mouth; these enlarge and coalesce to form milk-white superficial patches of considerable extent, and in time may cover the whole of the buccal cavity. It is usually accompanied by febrile and gastric disturbance. The disease is not dangerous unless it occurs with or after exhausting diseases.

Treatment. Attention to hygiene and general health, relief of any gastric disturbance, and here nitric acid is valuable as a tonic. Mouth washes, and if the fungus has already colonized, it must be combated by applying alkalies, as it, in common with other bud fungi, does not flourish on alkaline media. (Repeated wiping out the mouth with a cloth dipped in a 5-10 per cent. solution of bicarbonate of soda). In more serious cases hourly painting with $Ag\,No_3$ (0.1: 20 to 50). Internally chlorate of potassium (1:100.0 a teaspoonful every two hours). and where the œsophagus threatens to become obstructed—emetics.

VIII. Syphilitic Stomatitis.—This form must on no account be lightly passed over especially as it may lead to serious consequences both to the patient, family, physician and dentist. The syphilitic lesions of the mouth are—(1) those of the early secondary period, a transient erythema, mucous tubercles and superficial sores resulting from the latter and (2) the gummatous infiltrations of the latter stages, which tend to break down into ulcers. The affections may assume special characters in the tongue and palate. An erythema of the mouth and throat is commonly associated with the earliest cutaneous eruptions, but usually of short duration and requires no special treatment. Mucous tubercles show themselves in the early months of the disease. They appear as flat or slightly prominent, opaque, greyish patches, which by detachment of the affected epithelium may become converted into irregular excoriations. The points attacked are the angles of the mouth, tongue, tonsils, pillars of the fauces, but the lesion may, and often does, involve any portion of the buccal membrane. This affection, so quickly amendable to treatment in most other situations, is often very obstinate in the mouth. Why? Possibly owing to the continued irritation kept up by the normal and abnormal secretions, by the passage of food, by constant movements of the affected parts in speech, mastication and deglutition. The best local measures of treatment are scrupulous cleanliness, careful selection of diet and occasional applications of $Ag.No_3$ in stick or solutions.

The Gummata Syphiloma.—For convenience I divide them roughly into "superficial" and "deep." The first, confined to the mucous membrane, the latter, extending into the subjacent tissues. In any case the lesion, as a rule, is primarily manifested by a tubercular prominence, but may sometimes assume a more diffused character, as in "sclerous glossitis," of Fournier.

The gummata are always destructive in tendency and may lead to deep ulceration in the soft parts, or to necrosis where the bony walls of the mouth are attacked, and leave permanent and often unsightly scars.

The diagnosis of these syphilitic ulcers of the mouth is of more particular interest when the tongue is the seat of disease, but the distinction of the syphilitic ulcerations from epithelioma must be born in mind, in the investigation of all parts of the oral cavity. It is not easy to diagnose between malignant and gummatous ulcers. But, as a rule, the tertiary ulcer is marked by the absence of glandular enlargement, the comparatively slight induration of its base and borders, relative freedom from pain, its frequent localization in situations not exposed to direct irritation, by carious teeth, etc., and by the age and history of the patient. But age, history and locality in certain cases fail to afford any guiding indications. A syphilitic sore is occasionally painful and indurated, and there may be some irritative enlargement of the submaxillary glands, while on the other hand, an epithelioma in its early stages may cause no suffering, and may be long unassociated with any perceptible glandular implication. In cases of doubt the examination of a fragment taken from the surface of the ulcer and the tentative administration of rapidly increasing doses of iodide of potassium will often provide a solution to the problem ; or the sore may be scraped with a curette, a plan that may enable the surgeon to arrive at a positive conclusion without prejudice to further treatment. It must, however, be remembered that a syphilitic lesion is not an infrequent nidus for the development of malignant disease, and that it is hence unadvisable to temporize too long with a suspicions ulcer that resists all the appropriate treatment for the milder affection. The constitutional treatment of the later syphilitic diseases of the mouth is that of syphilis in general. Locally there is little to be done beyond the exercise of the precautions mentioned in connection with mucous tubercle. In concluding, it must be remembered that to treat a disease does not necessarily require that we should know the cause, yet, if we do happen to be so fortunate we can the more scientifically treat the disease, and here we must remember the great part that dental or oral antisepsis has to play in attaining that end and yet in spite of your antiseptics, without a good constitution and general health the micro-organisms and diseases will still develop. Here then we see the necessity of a dental surgeon being able to recognize these conditions and to aid in their treatment as well as his brother professional, the physician, to whom he should be closely allied and equal to, if not entirely combined with for the good of mankind.

The following works have been consulted in writing the above article.

1. Ashby and Wright, Diseases of Children.
2. Henoch, Lectures on Diseases of Children.
3. Samelsohn, Centralzeitung f. Kinderheilk (1) 1876. S. 190.
4. Sansone. Med. Chir. Trans. 1878.
5. Grawitz, Weber die Parasiten des Soors. etc. Virchow's Archiv. 1886. Bd. Ciii S. 393.
6. Miller, W. D. The Micro-Organisms of the Human Mouth.
7. Bristowe S.
8. Roberts, F.
9. Meigs and Pepper.
10. Tome's Dental Surgery.
11. Garretson " "
12. Harris " "
13. J. W. White, Dental Cosmos and article " Dentition."
14. Fraenkel. Transl. by Linsley. Bacteriology.
15. Crookshank. Bacteriology.
16. Fagge Principles and Practice of Medicine.
17. Payne. General Pathology.
18. Asburner J. On Dentition. 1834. (England.)
19. White, J. W. Dentition and Pathology of—Dental Cosmos—American System of Dentistry.
20. Pierre Dr. Marie Trans., by Del Valle 91. Stomatitis. June Dental Register. 91.
21. The Necessity for Constitutional Treatment in Dental Practice, Dr. Clifford, Dental Cosmos, 878. Oct. 1891.

